# Similarities in clinical course and outcome between juvenile idiopathic arthritis (JIA)-associated and ANA-positive idiopathic anterior uveitis: data from a population-based nationwide study in Germany

**DOI:** 10.1186/s13075-020-02166-3

**Published:** 2020-04-15

**Authors:** Arnd Heiligenhaus, Jens Klotsche, Martina Niewerth, Gerd Horneff, Gerd Ganser, Johannes-Peter Haas, Kirsten Minden

**Affiliations:** 1 Department of Ophthalmology at St. Franziskus Hospital, Muenster, Hohenzollernring 74, 48145 Muenster, Germany; 2 University of Duisburg-Essen, Duisburg, Germany; 3 German Rheumatism Research Center, a Leibniz Institute, Berlin, Germany; 4 Charité – University Medicine Berlin, Institute for Social Medicine, Epidemiology and Health Economics, Berlin, Germany; 5 Asklepios Clinic Sankt Augustin GmbH, Sankt Augustin, Germany; 6 Department of Pediatric and Adolescent Medicine, Medical Faculty, University Hospital of Cologne, Cologne, Germany; 7 Sankt Josef-Stift Sendenhorst, Sendenhorst, Germany; 8 German Centre for Pediatric and Adolescent Rheumatology, Garmisch-Partenkirchen, Germany; 9 Department of Rheumatology and Clinical Immunology, Charité - University Medicine Berlin, Berlin, Germany

**Keywords:** Complications, Disease-modifying anti-rheumatic drugs, Juvenile idiopathic arthritis, Population-based study, Uveitis

## Abstract

**Background:**

To analyze whether ANA-positive idiopathic anterior uveitis differs from JIA-associated uveitis concerning clinical course, response to treatment, and disease outcome.

**Methods:**

Prospective study of the National Paediatric Rheumatological Database (NPRD) including its uveitis add-on module from the years 2002 to 2016. Cross-sectional data from the years 2002 to 2016 were analyzed. Patients with JIA-associated uveitis and with ANA-positive idiopathic anterior uveitis were included and the disease manifestation investigated in terms of uveitis characteristics and disease course.

**Results:**

Of the total cohort of 34,458 patients enrolled in the NPRD, including 3551 patients with uveitis, those with detailed uveitis documentation were taken into account: 62 ANA-positive patients with idiopathic anterior uveitis (group 1), 688 patients with initial uveitis diagnosis after JIA onset (group 2), and 61 JIA patients with initial uveitis diagnosis before arthritis onset (group 3). Anterior uveitis was documented in 100%, 94%, and 80% of patients and with insidious onset of uveitis flare in 50%, 70.9%, and 56.1% each in groups 1, 2, and 3, respectively. Use of topical or systemic corticosteroids and conventional synthetic or biological DMARDs did not significantly differ between the patient groups, either at the initial or the 2-year follow-up (2-FU) visits (mean 2 years, each *p* > 0.05). At 2-FU, uveitis inactivity was achieved in 64.7%, 55.8%, and 61.5% of patients in groups 1, 2, and 3 (*p* > 0.05). Uveitis-related complications were more frequent at the initial visit and at 2-FU in groups 1 and 3, as compared to group 2.

**Conclusions:**

ANA-positive idiopathic uveitis and JIA-associated uveitis do not significantly differ concerning clinical course of uveitis, treatment, and response to corticosteroids and DMARDs.

## Background

Juvenile idiopathic arthritis (JIA) is a chronic inflammatory disease representing a heterogeneous group of chronic arthritis diseases with age at onset < 16 years, lasting for at least 6 weeks, and not attributed to other arthritic entities such as Lyme disease or septic or reactive arthritis or to systemic diseases such as lupus, vasculitis syndromes, or Crohn’s disease [1]. Incidence has been estimated at 3–23/100,000 children and prevalence of 16–140/100,000 [2–4]. In 9–13% of cases, uveitis occurrence has been documented [5–8], frequently causing eye complications and threatening vision [9–11]. Recent studies have determined that oligoarticular JIA, young age at onset, short duration of disease, and presence of antinuclear antibodies (ANA) are risk factors for the development of uveitis in JIA patients [6, 12–14].

Treatment for uveitis is mostly instituted with topical corticosteroids, and an escalation of therapy to disease-modifying anti-rheumatic drugs (DMARDs) is required in patients not responding properly or who present a particularly severe disease course [15, 16]. Population-based studies recently determined that changes in treatment patterns, i.e., more frequent use of DMARDs, reduced uveitis prevalence [17], and improved outcome of uveitis [8, 18]. Most recently, adalimumab has received approval for the treatment of chronic, noninfectious anterior uveitis in children aged ≥ 2 years either inadequately responding or not tolerating conventional therapy [19].

It is not well defined whether ANA-positive idiopathic anterior uveitis can be treated like JIA-associated uveitis and whether responses to treatment are similar. In a recent therapy guideline, it was accepted that uveitis can take a very similar course in patients with JIA and those with ANA positivity but no JIA, and that these two groups should be treated in the same way [16]. This is in accordance with the recently published CARRA consensus treatment plans for anterior uveitis [20], but this concept relies on previous observations from a single, retrospective, monocentric study and with a limited number of patients [21].

Current data on the occurrence and course of JIA are based on the International League of Associations for Rheumatology (ILAR) criteria [1]. Studies from tertiary uveitis and rheumatology centers may have been profoundly different owing to selection criteria and follow-up. Therefore, the data from the prospective Nationwide Paediatric Rheumatologic Database (NPRD) on children and adolescents with juvenile rheumatic diseases and with additional uveitis documentation in Germany have now been analyzed concerning the characteristics of JIA-associated uveitis and ANA-positive uveitis without associated arthritis (ANA-positive uveitis) in order to examine whether the disease characteristics and courses are similar.

## Methods

Phenotypes, clinical data, and outcomes from children and adolescents with juvenile rheumatic diseases are recorded once a year by pediatric rheumatologists from 62 participating centers within the prospective NPRD by a standardized case report form [22]. Incident and prevalent cases of juvenile rheumatic diseases are consecutively enrolled in the NPRD by all participating centers. The NPRD can be considered as representative data regarding clinical characteristics, as well as treatment assignments of children and adolescents with rheumatic diseases in Germany. The NPRD does not include an active study-monitoring and the yearly re-documentation rate is approximately 60%.

This analysis is based on patients who were recorded in the NPRD between January 1, 2002, and December 31, 2016. We selected patients with a diagnosis of JIA according to the ILAR criteria [1] to define the group of patients with JIA-associated uveitis (*n* = 3246). The group of ANA-positive uveitis (*n* = 305) patients was defined by a diagnosis of uveitis and the presence of ANA-positivity for whom no JIA was ever documented between 2002 and 2016.

For patients with uveitis, the treating ophthalmologists were additionally requested to complete an add-on uveitis questionnaire (uveitis module) once a year to describe uveitis characteristics [6, 8, 17]. The response rate for the uveitis module was 22.5% (726 of 3246) and 20.3% (62 of 305) for patients with JIA-associated and ANA-positive uveitis, respectively. Sociodemographic and clinical variables between patients with and without a uveitis module were comparable except for age at documentation at which the two groups differed in mean by 1 year. We refer for more details about the study design to Heiligenhaus et al. [6, 8]. Subjects’ consent was obtained according to the declaration of Helsinki, and the design of the work conforms to the standards of care currently applied in Germany. The study was approved by the Ethics Committee of the Charité University Medicine Berlin and by the local Ethics Committees, as required.

Referring pediatric rheumatologists documented patients’ age, gender, JIA category, age at arthritis onset, and treatment, physicians´ global assessment of disease activity on a numerical rating scale (NRS 0–10), the number of joints with arthritis, and extraarticular manifestations (e.g., presence of uveitis). Current use of systemic anti-inflammatory treatment, including DMARDs, was reported as well as for the last 12 months by the pediatric rheumatologist. Further, the presence of antinuclear antibodies (ANA) according to local laboratory standard, HLA-B27 antigen, and rheumatoid factor (RF) were documented.

The additional uveitis module provides information on uveitis at the first visit to the attending ophthalmologist (first documentation at or close to the initial uveitis diagnosis, initial documentation in the registry) and the current status (follow-up documentation) with regard to age at first onset of uveitis (initial uveitis diagnosis), uni- or bilateral uveitis, uveitis activity and symptoms, initial and current best-corrected visual acuity (logMAR), anti-inflammatory therapy for uveitis, initial and current eye complications, and previous eye surgeries. Uveitis was anatomically classified as proposed by SUN standardization [23].

Uveitis characteristics and anti-inflammatory treatment were compared between patients with JIA-associated uveitis and ANA-positive idiopathic anterior uveitis based on the information recorded at the first documentation by the ophthalmologist after uveitis onset. The source of information on uveitis characteristics at follow-up was the current status of uveitis at the documentation. A patient with JIA-associated uveitis (initial diagnosis before/after JIA onset) was matched to a patient with ANA-positive idiopathic anterior uveitis with respect to the duration between initial uveitis diagnosis and the date of the current visit to the ophthalmologist to ensure a comparable follow-up period between the three groups. Univariable analysis of variance for continuously distributed variables and the chi-squared test for categorical variables were used to test for differences between the three groups. Post hoc test with a Scheffé (analysis of variance) or Bonferroni (chi-squared test) correction was conducted for pairwise group comparisons. Data were analyzed using SAS software (version 9.3; SAS Institute Inc., Cary, NC, USA).

## Results

### Study population

**Fig. 1:**
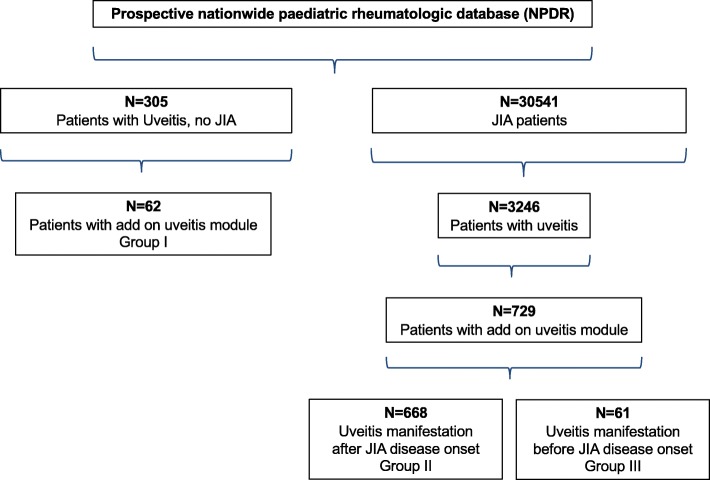
National Paediatric Rheumatological Database (NPRD) with a uveitis add-on module in Germany (2002–2016)

A total of 30,541 JIA patients were enrolled from 62 centers between 2002 and 2016 with the following JIA categories: persistent (*n* = 12,669) and extended oligoarthritis (*n* = 2228), rheumatoid factor (RF)-negative polyarthritis (*n* = 4854), and enthesitis-related arthritis (*n* = 4876, Table 1). Uveitis was documented in 3246 (10.6%) of JIA cases and occurred most frequently in patients with oligoarthritis (*n* = 2219, 14.9%). The data from those 729 JIA patients (22.5%) for whom the add-on uveitis module and documented course of uveitis data were available were included in the analysis (Fig. 1, Supplementary Table 1). They did not differ significantly from the other uveitis patients with respect to disease activity, the number of joints with arthritis, functional limitations (C-HAQ), gender, disease duration, and therapy.

**Table 1:** National Paediatric Rheumatological Database (NPRD) with a uveitis add-on module in Germany (2002–2016). Cumulative uveitis incidence in the different juvenile idiopathic arthritis (JIA) categories and in ANA-positive idiopathic anterior uveitis. Data as provided in NPRD and/or uveitis documentation

Data 2002–2016 subgroups	No. of patients with uveitis	Cumulative uveitis incidence rate; % [95% CI]	Median disease duration at uveitis onset*; years [IQR]
Systemic arthritis	42/1466	2.9 (2.1–3.8)	3.7 [1.4–7.8]
Oligoarthritis (persistent)	1792/12669	14.1 (13.6–14.8)	2.8 [1.1–5.6]
Oligoarthritis (extended)	427/2228	19.2 (17.6–20.8)	5.8 [3.0–9.8]
Polyarthritis (RF-negative)	359/4854	7.4 (6.7–8.2)	3.4 [1.5–6.5]
Polyarthritis (RF-positive)	19/672	2.8 (1.8–4.3)	3.3 [1.7–5.8]
Enthesitis-related arthritis	348/4876	7.1 (6.4–7.9)	2.7 [1.2–4.9]
Psoriatic arthritis	153/2316	6.6 (5.6–7.7)	3.3 [1.4–6.6]
Other arthritis	106/1420	7.5 (6.2–8.9)	2.5 [0.8–5.3]
All JIA patients	3246/30541	10.6 (10.3–11-0)	3.1 [1.3–6.1]
ANA-positive, no JIA	305		

*RF* rheumatoid factor, *IQR* interquartile range*

*Initial uveitis diagnosis

A total of 305 uveitis patients were identified who were ANA-positive but presented no JIA (Table 1). Among these, ophthalmologic data were available for 62 (20.3%, group 1) (Fig. 1). Notably, the data from these patients with a documented uveitis course did not differ from the other uveitis patients with respect to gender, age at disease onset, presence of ANA positivity, and therapy.

### Patient and uveitis characteristics at initial documentation

The demographic characteristics of JIA-associated and ANA-positive idiopathic uveitis patients at initial documentation are summarized in Table 2. The duration between JIA and initial uveitis diagnosis (24.2 vs. − 19.4 months) and age at JIA onset (4.2 vs. 7.1 years) significantly differed between patients with uveitis manifestation after (*n* = 688, group 2) and before arthritis onset (*n* = 61, group 3), respectively (*p* < 0.001).

**Table 2:** National Paediatric Rheumatological Database (NPRD) with a uveitis add-on module in Germany (2002–2016). Demographic characteristics. Data as provided in NPDR and/or uveitis documentation

Initial documentation. Demographic characteristics	Group I. Uveitis, ANA-positive, no JIA, *N* = 62			Group II. Uveitis manifestation after JIA disease onset*, *N* = 668			Group III. Uveitis manifestation before JIA disease onset*, *N* = 61			*p* value^†^	Significant pairwise comparisons^‡^
	*n*	Mean	SD	*n*	Mean	SD	*n*	Mean	SD		
Duration between JIA and uveitis onset, months (SD)	n.a.	n.a.	n.a.	668	24.2	32.8	61	-19.4	29.7	< 0.001	II vs. III
Age at JIA onset, years (SD)	n.a.	n.a.	n.a.	668	4.2	3.3	61	7.1	4.0	< 0.001	II vs. III
Age at uveitis onset, years (SD)	56	8.4	3.8	668	6.3	3.8	61	5.5	4.1	< 0.001	I vs. II, I vs. III
Duration between uveitis onset and initial documentation, months (SD)	56	20.2	23.1	641	57.5	50.8	59	36.5	33.3	< 0.001	I vs. II, II vs. III

*n.a.* not applicable

*Initial uveitis diagnosis

^†^Group effect by univariate analysis of variance

^‡^Pairwise comparisons with correction for multiple comparisons by Scheffé

Age at initial uveitis diagnosis did not significantly differ comparing the JIA patient groups 2 and 3, while it was increased in ANA-positive uveitis patients (*p* < 0.001). The duration between uveitis manifestation and first uveitis documentation was shorter in group 1 than in the other two groups (*p* < 0.001).

Uveitis characteristics at initial documentation are summarized in Table 3. The most common anatomic type of uveitis was anterior uveitis in all patient groups. Onset of flare was insidious in more than two thirds of JIA patients in whom uveitis became manifest after JIA onset. However, a sudden (acute, symptomatic) onset of flare was observed in about 1/3 of patients with ANA-positive uveitis and with uveitis onset before onset of JIA.

**Table 3:** National Paediatric Rheumatological Database (NPRD) with a uveitis add-on module in Germany (2002–2016). Uveitis characteristics at initial documentation. Data as provided in uveitis documentation

Initial documentation. Uveitis characteristics	Group I. Uveitis, ANA-positive, no JIA, *N* = 62			Group II. Uveitis manifestation after JIA disease onset*, *N* = 668			Group III. Uveitis manifestation before JIA disease onset*, *N* = 61			*p* value^†^	Significant pairwise comparisons^‡^
	*N* (%)	Mean	SD	*N* (%)	Mean	SD	*N* (%)	Mean	SD		
Anterior uveitis, *n* (%)	62 (100)	n.a.	n.a.	546 (94.0)	n.a.	n.a.	40 (80.0)	n.a.	n.a.	< 0.001	I vs. II, I II vs. III
Insidious onset of flare, *n* (%)	25 (50.0)	n.a.	n.a.	363 (70.9)	n.a.	n.a.	23 (56.1)	n.a.	n.a.	0.002	I vs. II
Bilateral uveitis, *n* (%), data provided in 599 cases	33 (67.3)	n.a.	n.a.	241 (48.9)	n.a.	n.a.	28 (59.6)	n.a.	n.a.	0.015	I vs. II
Active uveitis (AC cell grade ≥ 1+); *n* (%)	26 (66.7)	n.a.	n.a.	191 (53.8)	n.a.	n.a.	13 (61.9)	n.a.	n.a.	0.316	–
Visual acuity in uveitis eyes, logMAR	74	0.35	0.36	701	0.23	0.37	59	0.38	0.47	0.020	–
Worse eyes, logMAR	61	0.44	0.49	612	0.28	0.42	55	0.42	0.55	0.038	I vs. II

^†^Group effect by univariate analysis of variance for continuously distributed variables and chi-squared test for categorical variables

^‡^Pairwise comparisons with correction for multiple comparisons by Scheffé (analysis of variance) or Bonferroni (chi-squared test)

*Initial uveitis diagnosis

In all groups, more than half of the patients showed bilateral eye involvement, being significantly more common in patients with ANA-positive uveitis than in those with initial uveitis diagnosis after JIA disease onset. More than half of the patients in all patient groups (53.8–66.7%) had active uveitis (AC cell grade > 0.5+) at initial documentation in the study, with the percentage being slightly higher in patients of group 1 (66.7%) and group 3 (61.9%). Mean logMAR visual acuity was worse in patients with ANA-positive uveitis and when uveitis had become manifest before JIA onset (*p* < 0.05) as compared to patients in whom uveitis became manifest after arthritis onset (Table 3).

In 72.9% of patients with ANA-positive uveitis and in 66.1% of patients with uveitis that had become manifest before JIA onset, uveitis complications were already noted at the initial uveitis documentation (Table 4). Correspondingly, particular complications typically related to uveitis (e.g., synechiae and cataract formation) developed more frequently in these two patient groups. In contrast, uveitis-related complications were less common (39.9%) in patients in whom uveitis became manifest (initial uveitis diagnosis) after JIA disease onset (*p* < 0.001).

**Table 4:** National Paediatric Rheumatological Database (NPRD) with a uveitis add-on module in Germany (2002–2016). Uveitis-related eye complications at initial documentation. Data as provided in uveitis documentation

Initial documentation. Uveitis-related eye complications at initial documentation, per patient, *n* (%)	Group I. Uveitis, ANA-positive, no JIA, *N* = 62		Group II. Uveitis manifestation after JIA disease onset*, *N* = 668		Group III. Uveitis manifestation before JIA disease onset*, *N* = 61		*p* value^†^	Significant pairwise comparisons^‡^
	*N*	%	*N*	%	*N*	%		
Any complication	43	72.9	252	39.9	37	66.1	< 0.001	I vs. II, II vs. III
Band keratopathy	7	11.9	53	8.4	9	16.1	0.126	–
Glaucoma	2	3.4	10	1.6	5	8.9	0.002	II vs. III
Synechiae	29	49.2	184	29.1	25	44.6	< 0.001	I vs. II, II vs. III
Ocular hypotony	0	0.0	4	0.6	1	1.8	0.482	–
Retinal detachment	1	1.7	0	0.0	1	1.8	0.004	I vs. II, II vs. III
Papille edema	2	3.4	37	5.9	5	8.9	0.449	–
Iris rubeosis	3	5.1	12	1.9	1	1.8	0.266	–
Cataract	15	25.4	73	11.6	14	25.0	0.004	I vs. II, II vs. III
Vitreous opacities	13	22.0	41	6.5	6	10.7	< 0.001	I vs. II
Macular edema	4	6.8	22	3.5	3	5.4	0.382	–
Amblyopia/strabism	3	5.1	17	2.7	3	5.4	0.352	–
Other complications	3	5.1	21	3.3	3	5.4	0.604	–
Treatment-related eye complications	1	1.7	3	0.5	1	1.8	0.309	–

^†^Group effect by chi-squared test for categorical variables

^‡^Pairwise comparisons with correction for multiple comparisons by Bonferroni (chi-squared test)

*Initial uveitis diagnosis

The anti-inflammatory treatment and achievement of uveitis inactivity at initial documentation are summarized in Table 5. In up to 2/3 of patients, DMARD use in the previous 12 months was noted. Compared to ANA-positive uveitis patients, those with JIA-associated uveitis (both with uveitis manifestation before and after arthritis onset) had been treated more often with methotrexate and less frequently with systemic corticosteroids. Adalimumab (< 10%) and topical corticosteroids (approximately 60%) were used at comparable frequencies in these groups. Compared to the other 2 groups, uveitis inactivity was documented more often with DMARD use (28.0%) in patients in whom uveitis became manifest after JIA disease onset.

**Table 5:** National Paediatric Rheumatological Database (NPRD) with a uveitis add-on module in Germany (2002–2016). Initial uveitis documentation: anti-inflammatory treatment in previous 12 months. Data as provided in NPDR and/or uveitis documentation

Initial documentation. Anti-inflammatory treatment in previous 12 months	Group I. Uveitis, ANA-positive, no JIA, *N* = 62		Group II. Uveitis manifestation after JIA disease onset*, *N* = 668		Group III. Uveitis manifestation before JIA disease onset*, *N* = 61		*p* value^†^	Significant pairwise comparisons^‡^
	Treatment	Uveitis inactivity^‡‡^	Treatment	Uveitis inactivity^‡‡^	Treatment	Uveitis inactivity^‡‡^		
Any DMARD, *n* (%)	31 (52.5%)	2 (9.5%)	424 (65.6%)	63 (28.0%)	42 (70.0%)	1 (6.7%)	0.091	–
Methotrexate, *n* (%)	27 (45.8%)	1 (5.3%)	382 (59.1%)	59 (29.1%)	40 (66.7%)	1 (7.1%)	0.058	I vs. III
Adalimumab, *n* (%)	5 (8.5%)	1 (20.0%)	41 (6.4%)	8 (26.7%)	4 (6.7%)	0 (0.0%)	0.817	–
Systemic corticosteroids, *n* (%)	18 (29.0%)	1 (10.0%)	128 (19.3%)	22 (35.5%)	16 (26.2%)	1 (25.0%)	0.108	–
Topical corticosteroids, *n* (%)	36 (58.1%)	5 (19.2%)	388 (58.2%)	53 (23.5%)	37 (60.7%)	1 (8.3%)	0.930	–

^†^Group effect for the proportion of treated patients by chi-squared test for categorical variables

^‡^Pairwise comparisons for the proportion of treated patients with correction for multiple comparisons by Bonferroni (chi-squared test)

^‡‡^Group I: *n* = 39; Group II: *n* = 355; Group III: *n* = 21 patients with documented AC cell count

*Initial uveitis diagnosis

### Patient and uveitis characteristics at follow-up documentation

For comparison of data during the further uveitis course, a mean follow-up of 2 years (2-FU) after initial uveitis diagnosis could be analyzed (Table 6). Hence, bilateral manifestation of uveitis was significantly more frequent in ANA-positive uveitis than in patients in whom uveitis became manifest after JIA disease onset, whereas the numbers in groups 2 and 3 did not differ significantly from each other. Inactive uveitis according to the SUN guideline (AC cell grade < 0.5+) was achieved at quite similar levels (55.8% to 64.7%) in the three patient groups.

**Table 6:** National Paediatric Rheumatological Database (NPRD) with a uveitis add-on module in Germany (2002–2016). Uveitis characteristics at follow-up documentation. Data as provided in uveitis documentation

Follow-up documentation. Uveitis characteristics	Group I. Uveitis, ANA-positive, no JIA, *N* = 56			Group II. Uveitis manifestation after JIA disease onset*, *N* = 56			Group III. Uveitis manifestation before JIA disease onset*, *N* = 37			*p* value^†^	Significant pairwise comparisons^‡^
	*N* (%)	Mean	SD	*N* (%)	Mean	SD	*N* (%)	Mean	SD		
Duration of uveitis onset until follow-up documentation, months	56	20.2	23.1	56	20.2	23.1	37	19.7	18.0	0.992	–
Unilateral uveitis, *n* (%), data provided in 110 cases	11 (27.5)	n.a.	n.a.	25 (61.0)	n.a.	n.a.	13 (44.8)	n.a.	n.a.	0.035	I vs. II
Bilateral uveitis, *n* (%), data provided in 110 cases	29 (72.5)	n.a.	n.a.	16 (39.0)	n.a.	n.a.	14 (55.2)	n.a.	n.a.		
Inactive uveitis (AC cell grade < 0.5+); *n* (%), data provided in 74 cases	22 (64.7)	n.a.	n.a.	15 (55.8)	n.a.	n.a.	8 (61.5)	n.a.	n.a.	0.854	–
Visual acuity in uveitis eyes, logMAR	64	0.28	0.31	53	0.24	0.41	37	0.38	0.51	0.152	–
Worse eyes, logMAR	52	0.31	0.46	53	0.29	0.52	33	0.47	0.58	0.038	I vs. III, II vs. III

*n.a.* not applicable, *n.s.* not significant

*Initial uveitis diagnosis

^†^Group effect for the proportion of treated patients by chi-squared test for categorical variables

^‡^Pairwise comparisons for the proportion of treated patients with correction for multiple comparisons by Bonferroni (chi-squared test)

At 2-FU, mean and worse-eye logMAR visual acuities were the poorest in patients in whom uveitis became manifest (initial uveitis diagnosis) before JIA disease and ANA-positive uveitis onset and were significantly better in patients in whom uveitis was initially diagnosed after JIA developed (*p* = 0.038, Table 6). As compared to the initial documentation, mean logMAR improved slightly in ANA-positive uveitis and in patients in whom uveitis became manifest after JIA disease onset. In patients in whom uveitis was initially diagnosed before JIA disease onset, however, the mean logMAR was unchanged and the worse-eye logMAR visual acuity deteriorated slightly.

The number of patients with uveitis-related eye complications (Table 7) at 2-FU had slightly dropped in patients with ANA-positive uveitis and in patients with initial uveitis diagnosis before JIA disease onset as compared to the initial documentation, e.g., for the inflammation-related variables synechiae, vitreous opacities, and macular edema. Yet, the complication rates were slightly higher in patients with ANA-positive uveitis without JIA (56.9%) or in whom uveitis was initially diagnosed before the onset of JIA (57.6%) than in the group with uveitis occurrence after JIA onset (37.7%) (*p* = 0.086).

**Table 7:** National Paediatric Rheumatological Database (NPRD) with a uveitis add-on module in Germany (2002–2016). Uveitis-related eye complications at follow-up documentation. Data as provided in uveitis documentation

Follow-up documentation. Uveitis-related eye complications per patient, *n* (%)	Group I. Uveitis, ANA-positive, no JIA, *N* = 56		Group II. Uveitis manifestation after JIA disease onset*, *N* = 56		Group III. Uveitis manifestation before JIA disease onset*, *N* = 37		*p* value^†^	Significant pairwise comparisons^‡^
	*N*	%	*N*	%	*N*	%		
Any complication	29	56.9	20	37.7	19	57.6	0.086	
Band keratopathy	7	13.7	2	3.8	9	27.3	0.072	II vs. III
Glaucoma	4	7.8	0	0.0	0	0.0	0.031	
Synechiae	16	31.4	13	24.5	11	33.3	0.622	
Ocular hypotony	0	0.0	0	0.0	2	6.1	0.041	
Retinal detachment	1	2.0	0	0.0	1	3.0	0.500	
Papille edema	3	5.9	2	3.8	2	6.1	0.852	
Iris rubeosis	3	5.9	0	0.0	1	3.0	0.205	
Cataract	10	19.6	5	9.4	6	18.2	0.309	
Vitreous opacities	5	9.8	4	7.6	2	6.1	0.816	
Macular edema	2	3.9	2	3.8	1	3.0	0.976	
Amblyopia/strabism	3	5.9	0	0.0	2	6.1	0.194	
Other complications	2	3.9	5	9.4	3	9.1	0.503	
Treatment-related eye complications	1	2.0	1	1.9	1	3.0	0.931	

^†^Group effect for the proportion of treated patients by chi-squared test for categorical variables

^‡^Pairwise comparisons for the proportion of treated patients with correction for multiple comparisons by Bonferroni (chi-squared test)

*Initial uveitis diagnosis

The use of DMARDs and corticosteroids and the achievement of uveitis inactivity at 2-FU are summarized in Table 8. Although a slightly more frequent use of DMARDs and corticosteroids was documented in patients in whom uveitis was initially diagnosed before JIA onset than in the other two patient groups, the numbers did not differ significantly between the three groups. Hence, the number of patients with documented achievement of uveitis inactivity roughly corresponded with this notion.

**Table 8:** National Paediatric Rheumatological Database (NPRD) with a uveitis add-on module in Germany (2002–2016). Follow-up uveitis documentation: anti-inflammatory treatment in previous 12 months. Data as provided in NPDR and/or uveitis documentation

Follow-up documentation. Anti-inflammatory treatment in previous 12 months	Group I. Uveitis, ANA-positive, no JIA, *N* = 56		Group II. Uveitis manifestation after JIA disease onset*, *N* = 56		Group III. Uveitis manifestation before JIA disease onset*, *N* = 37		*p* value^†^	Significant pairwise comparisons^‡^
	Treatment	Uveitis inactivity^‡‡^	Treatment*	Uveitis inactivity^‡‡^	Treatment	Uveitis inactivity^‡‡^		
Any DMARD, *n* (%)	29 (53.7%)	13 (61.9%)	27 (49.1%)	5 (45.5%)	23 (63.9%)	6 (66.7%)	0.379	
Methotrexate, *n* (%)	25 (46.3%)	11 (61.1%)	26 (47.3%)	5 (50.0%)	23 (63.9%)	6 (66.7%)	0.204	
Adalimumab, *n* (%)	5 (12.2%)	3 (60.0%)	4 (12.9%)	2 (66.7%)	2 (9.5%)	0 (0.0%)	0.805	
Systemic corticosteroids, *n* (%)	16 (32.7%)	6 (54.6%)	7 (13.2%)	1 (100.0%)	12 (35.3%)	2 (50.0%)	0.044	II vs. III
Topical corticosteroids, *n* (%)	33 (62.3%)	13 (54.2%)	29 (59.2%)	8 (50.0%)	24 (70.6%)	2 (28.6%)	0.446	

^†^Group effect for the proportion of treated patients by chi-squared test for categorical variables

^‡^Pairwise comparisons for the proportion of treated patients with correction for multiple comparisons by Bonferroni (chi-squared test)

^‡‡^Group I: *n* = 34; Group II: *n* = 27; Group III: *n* = 13 patients with documented AC cell count

*Initial uveitis diagnosis

## Discussion

Data presented herein emphasize the similarities between JIA-associated uveitis and ANA-positive idiopathic uveitis in childhood and adolescence concerning demographic characteristics, clinical appearance of uveitis, occurrence of complications, need for corticosteroids and DMARDs, and achievement of uveitis inactivity.

A previous study from Holland et al. [21] reported on a retrospective case series from a single center examined by one author and included patients with anterior and intermediate uveitis as per SUN criteria [23]. From a total of 115 patients with chronic anterior uveitis (in 200 eyes), 44 had associated JIA and another 2 sarcoidosis. When children with JIA were compared to the others without systemic disease, the patient groups did not differ concerning vision-threatening complications at baseline examination, including, for example, band-keratopathy, peripheral posterior synechiae, cataract, inflammatory pupillary and ciliary membranes, glaucoma/elevated IOP, hypotony, and macular edema. In addition, associated JIA disease did not represent a significant risk factor for vision loss or for the development of vision-threatening complications during the follow-up (median, 23.5 months) in 148 eyes from 83 children with uveitis.

Our study employed a prospective, population-based, nationwide database [6, 8, 17], including a total of more than thirty thousand patients. We applied the current ILAR classification and SUN criteria [1, 23], and patients were included only if they had anterior uveitis typical for JIA-associated uveitis [24], also including ANA-positive uveitis children without JIA.

Compared with previous studies on JIA-associated uveitis, our study population is consistent with previous observations concerning age at onset, gender, uveitis occurrence in JIA subgroups, ANA positivity, and uveitis characteristics [6, 8, 13, 25, 26], which underscores the valuable and representative data within this study.

An important result of this study is that ANA-positive idiopathic anterior uveitis does not differ from JIA-associated uveitis in terms of uveitis characteristics, clinical course, and requirement for anti-inflammatory treatment and use of DMARDs. There were no major significant differences between idiopathic uveitis and JIA-associated uveitis diagnosed before arthritic symptoms. The frequent occurrence of vision-threatening eye complications here is probably related to the fact that, in the absence of joint problems and related morbidity, patients were not aware of ensuing ocular disease and did not consider screening guidelines as for JIA, except for detecting amblyopia as recommended by the minister of health and professional public health authorities.

The data provided confirm the common asymptomatic course of insidious-onset uveitis in the white globe in many of the JIA patients suffering from oligoarthritis, RF-negative polyarthritis, unclassified arthritis, and psoriatic arthritis. The observations also confirm the higher complication rates of early-onset uveitis, with eye manifestation before arthritis onset [6]. The complication rates—notably, approximately one third of patients at initial diagnosis—are in line with previous reports on JIA uveitis [6, 25, 27].

The observations made in this study substantiate ANA-positive idiopathic uveitis not to differ from JIA-associated uveitis regarding uveitis-related symptoms and that even higher complication rates are found at initial diagnosis than for JIA uveitis. Whereas in JIA patients, risk markers of JIA uveitis, including ANA positivity, young age at arthritis onset, and oligoarticular disease [6, 13], may help to determinate patients at high risk for uveitis subsequently introducing uveitis screening and early treatment; these caveats obviously cannot be applied in patients with ANA-positive uveitis without arthritis. Thus, diagnosis cannot be made before eye complications are present. Indeed, ANA-positive uveitis was initially diagnosed significantly later in age than in the JIA patients. Conversely, about one half of patients with ANA-positive idiopathic uveitis and also those with initial uveitis diagnosis before JIA disease onset developed an acute onset of flare, which probably led to earlier detection of eye disease and to proper treatment.

Notably, nearly two thirds of patients with ANA-positive uveitis without JIA or uveitis preceding the onset of JIA in this cohort had already developed typical uveitis-related complications at the initial visit, which again was probably related to the asymptomatic uveitis course in these patients. The high percentage of ocular complications confirms previous reports [6, 10, 28]. In contrast, uveitis-related complications were less common (39.9%) in patients in whom uveitis was initially diagnosed after the onset of JIA (*p* = 0.001), probably owing to early recognition of disease, institution of DMARD treatment, and also reduced use of corticosteroids. Indeed, recent publications reported that complications rates in JIA uveitis were reduced under early use of DMARDs [17].

JIA uveitis patients in our study frequently were on DMARDs at initial documentation and at 2-FU, which is attributed to the severity of this type of uveitis [9, 17, 29]. The use of systemic corticosteroids and DMARDs was also frequent in ANA-positive idiopathic uveitis, which confirms recent data from the CARRA registry on medication use in juvenile uveitis patients [30].

Uveitis inactivity was achieved similarly frequent in JIA uveitis and ANA-positive idiopathic uveitis patients and was observed after adjusting DMARD treatment more often at 2-FU (see data in Tables 5 and 8) than at initial documentation.

The 2-FU visual acuity in ANA-positive idiopathic uveitis patients was not significantly better than in the JIA group. Further, it was worst in patients with early manifestation of uveitis before arthritis, supporting previous notions in early-onset JIA uveitis patients [6, 10, 29, 31]. Indeed, visual outcome is highly influenced by the complicated uveitis course prior to diagnosis and before instituting treatment. Furthermore, in a recent study analyzing the outcomes of noninfectious pediatric uveitis, visual outcomes in JIA-associated and idiopathic uveitis cohorts were not significantly different [32].

In agreement with previous assumptions, poor vision generally correlated with presence of ocular complications. The type of complications and their frequencies found herein are typical for JIA uveitis and agree with previous reports [6, 8, 11, 28, 29]. Notably, the respective figures were not different from those in ANA-positive idiopathic uveitis patients.

Although this large, prospective, population-based analysis of more than 3500 JIA- and ANA-positive patients with uveitis was conducted according to currently accepted, standardized methods of detailed rheumatological and ophthalmological documentation [23, 33], the NPRD also has certain limitations. While patients were followed up for more than 10 years, data on 2-FU were chosen for proper comparability of the groups. Each patient was only documented once a year for the entire study population and during that period so that this had no impact on the group characteristics illustrated. Indeed, a detailed ophthalmological dataset on uveitis characteristics, secondary complications, and anti-inflammatory treatment was only completed for about one fifth of uveitis patients, but the study groups were representative for important patient- and disease-related characteristics. In addition, the NPRD does not include an active study-monitoring resulting in a lower number of patients in the 2-year follow-up assessment. This may introduce a risk for selection bias toward patients with a more severe uveitis. Furthermore, median disease duration in the ANA-positive patients herein is shorter than in the JIA groups, not entirely excluding the risk for subsequent arthritis manifestation.

In conclusion, the data from the prospective, nationwide NPRD with add-on uveitis module in Germany show that uveitis in ANA-positive children without arthritis appears to take a course similar to that of uveitis in patients with JIA and thus should be managed similarly. Early detection of ANA, ESR, or S100 proteins in the general population—although they would relate to any systemic inflammatory process—may further simplify early detection of children at risk of uveitis.

## Conclusions

ANA-positive uveitis without arthritis and JIA-associated uveitis did not differ regarding uveitis characteristics and complications, treatment with corticosteroids and DMARDs, and response to treatment.

## Supplementary information

**Additional file 1 Supplementary Table 1.** National Paediatric Rheumatological Database (NPRD) with a uveitis add-on module in Germany (2002-2016). Uveitis manifestation in the diverse categories of juvenile idiopathic arthritis. Data as provided in NPDR. n.a. = not applicable; * initial uveitis diagnosis.

## Data Availability

The study was approved by the Ethics Committee of the Charité University Medicine Berlin and by the local Ethics Committees, as required.

## Abbreviations

ANA: Antinuclear antibodies
DMARDs: Disease-modifying anti-rheumatic drugs
FU: Follow-up
ILAR: International League of Associations for Rheumatology
IOP: Intraocular pressure
JIA: Juvenile idiopathic arthritis
NPRD: National Paediatric Rheumatological Database
NRS: Numerical rating scale
RF: Rheumatoid factor
SUN: Standardization of uveitis nomenclature
