# Supplementary material for: Similarities in clinical course and outcome between juvenile idiopathic arthritis (JIA)-associated and ANA-positive idiopathic anterior uveitis: data from a population-based nationwide study in Germany
Source: Arthritis Res Ther. 2020 Apr 15;22:81. doi: 10.1186/s13075-020-02166-3 (PMC7161187; doi:10.1186/s13075-020-02166-3)
Supplement: Supplementary file 1 — Additional file 1 Supplementary Table 1. National Paediatric Rheumatological Database (NPRD) with a uveitis add-on module in Germany (2002-2016). Uveitis manifestation in the diverse categories of juvenile idiopathic arthritis. Data as provided in NPDR. n.a. = not applicable; * initial uveitis diagnosis. [file 13075_2020_2166_MOESM1_ESM.docx]

**Supplementary Table 1.** National Paediatric Rheumatological Database (NPRD) with a uveitis add-on module in Germany (2002-2016). Uveitis manifestation in the diverse categories of juvenile idiopathic arthritis. Data as provided in NPDR.

| **Initial documentation.**  JIA category | Group I.  Uveitis, ANA positive, no JIA  N=62 | | Group II.  Uveitis manifestation after JIA disease onset*  N=668 | | Group III.  Uveitis manifestation  before JIA disease onset*  N=61 | | P-value |
| --- | --- | --- | --- | --- | --- | --- | --- |
|  | N | % | N | % | N |  | II vs III |
| Polyarthritis, RF positive | n.a. | n.a. | 3 | 0.5 | 1 | 1.6 | 0.082 |
| Polyarthritis, RF negative | n.a. | n.a. | 78 | 11.7 | 3 | 4.9 |  |
| Systemic arthritis | n.a. | n.a. | 10 | 1.5 | 1 | 1.6 |  |
| Oligoarthritis, persistent | n.a. | n.a. | 405 | 60.5 | 29 | 47.6 |  |
| Oligoarthritis, extended | n.a. | n.a. | 76 | 11.4 | 11 | 18.1 |  |
| Psoriatic arthritis | n.a. | n.a. | 16 | 2.4 | 4 | 6.6 |  |
| Enthesitis-related arthritis | n.a. | n.a. | 50 | 7.5 | 8 | 13.1 |  |
| Other arthritis | n.a. | n.a. | 30 | 4.5 | 4 | 6.5 |  |

n.a. = not applicable; * initial uveitis diagnosis
